# A Reference Handbook of the Medical Sciences

**Published:** 1901-10

**Authors:** 


A Reference Handbook of the Medical Sciences, embracing the entire range of scientific and practical medicine and allied science. By various writers. A new edition, completely revised and rewritten. Edited by Albert H. Buck, M. D., New York City. Volume II. Illustrated by numerous chromo-lithographs and seven hundred and sixty-five half-tone and wood engravings. Price per volume, cloth, $6.50; morocco, $7. New York: William Wood & Co. 1901.

This is one of the sets of books that will go back into the library of every scientific man who had access to the first edition. Specialization in books has reached the point where one is liable to forget where the little book is when he is in need of information on an off subject. A minister called on the writer a few days ago and requested the loan of a book that contained a scientific discussion of suicide. Out of a library of over a thousand volumes the Reference Handbook was the only place a decent article could be found. And so it is on all subjects outside of the most common place.

It is unnecessary to speak of the work with reference to the practical features for it is up to date and thoroughly revised.

Comparing Volume II, revised, with the same volume of the old edition one can appreciate the amount of work done and the improvements that show in the new edition.

This is a valuable work of reference, unequaled by any with which I am acquainted. T. J. B.

